# Coronary angiography in cardiac arrest patients undergoing extracorporeal cardiopulmonary resuscitation

**DOI:** 10.1007/s12471-026-02049-3

**Published:** 2026-05-18

**Authors:** Margriet Bogerd, Jarno N. R. de Haas, Sanne ten Berg, Luuk C. Otterspoor, Bimmer E. P. M Claessen, Niels J. W. Verouden, Marcel A. M. Beijk, Paul Knaapen, Yolande Appelman, Jan Baan, Jorrit S. Lemkes, Marije M. Vis, Alexander W. den Hartog, Maik J. Grundeken, Alexander Nap, Ronak Delewi, Alexander P. J. Vlaar, Annemarie E. Engström, José P. S. Henriques

**Affiliations:** 1 Department of Cardiology, Amsterdam UMC, Amsterdam, The Netherlands; 2 Department of Cardiology and Intensive Care, Catherina Hospital, Eindhoven, The Netherlands; 3 Department of Intensive Care, Amsterdam UMC, Amsterdam, The Netherlands

**Keywords:** Cardiac arrest, Extracorporeal Cardiopulmonary Resuscitation, VA-ECMO, Coronary Angiography, Electrocardiography, Coronary artery disease

## Abstract

**Background:**

The use of extracorporeal cardiopulmonary resuscitation (ECPR) is emerging. In cardiac arrest patients with return of spontaneous circulation (ROSC), the electrocardiogram (ECG) guides the need for immediate coronary angiography (CAG). In ECPR patients, the diagnostic value of the ECG and the role of CAG remain unclear.

**Methods:**

This single-centre, retrospective study included all adult ECPR patients admitted to Amsterdam UMC (2018–2024). ECPR was defined as cannulation on venoarterial extracorporeal membrane oxygenation during ongoing continuous or intermittent resuscitation, before sustained ROSC (≥ 20 min). The primary outcome included the presence and extent of coronary artery disease (CAD). Logistic regression was used to identify predictors of undergoing CAG and having a culprit. The impact of CAG on survival was assessed using Cox proportional hazard models and Kaplan-Meier curves.

**Results:**

Ninety-two patients (mean age of 53 years, 65% male) were included, with 70% presenting after out-of-hospital cardiac arrest and 52% with an initial shockable rhythm. Survival to discharge was 18%. Survivors more often had intermittent ROSC and were less often obese. CAG was performed in 59%, revealing significant CAD in 94%, multivessel disease in 60%, and a culprit lesion in 78%. Revascularisation occurred in 74%. Among patients with ST-segment elevations, those without CAG had higher in-hospital mortality (HR 4.06, 95%CI 1.72–9.61).

**Conclusion:**

The presence of CAD and culprit lesions was high among ECPR patients undergoing CAG. Early CAG was associated with a benefit in patients with ST-segment elevations. Further research is necessary to evaluate the benefit of early CAG in patients without ST-segment elevation.

**Supplementary Information:**

The online version of this article (10.1007/s12471-026-02049-3) contains supplementary material, which is available to authorized users.

## What’s new?

- This is the first Dutch cohort study to systematically evaluate the diagnostic value of the ECG and the role of coronary angiography in ECPR patients.
- Among ECPR patients undergoing CAG, 94% had significant coronary artery disease, 78% had a culprit lesion, 60% had multivessel disease and 26% had at least one CTO.
- The benefit of CAG seemed to differ by ECG findings, showing significant benefit in those presenting with ST-elevation. Further studies are needed to clarify the role of early CAG in those without ST-elevation.
- ECPR survivors showed higher rates of intermittent ROSC prior to cannulation and were less often obese.

## Introduction

Cardiac arrest (CA) is a devastating condition with poor survival rates [1, 2]. Most adult CA cases are linked to obstructive coronary artery disease (CAD) and acute myocardial infarction (AMI) [3, 4]. After return of spontaneous circulation (ROSC), further diagnostics are largely guided by the electrocardiogram (ECG), where immediate coronary angiography (CAG) is recommended for those presenting with ST-segment elevations, while immediate CAG is not beneficial in those without ST-segment elevation [4–8].

Extracorporeal cardiopulmonary resuscitation (ECPR) has recently emerged for patients who do not achieve sustained ROSC, creating a new subset of CA patients. An initial meta-analysis, including propensity-matched cohort studies, demonstrated improved survival and neurological outcome with ECPR compared to conventional cardiopulmonary resuscitation (CCPR) [9]. Subsequent randomised controlled trials (RCTs) have yielded divergent results [10–12]. However, pooled patient-level analyses using both frequentist and Bayesian approaches suggest that ECPR improves survival with good neurological outcome, particularly in patients with witnessed CA and shockable rhythms, and may thus be an effective treatment for selected patients in highly specialised, multidisciplinary systems [13–16].

ECPR enables additional diagnostic and therapeutic interventions in those with refractory CA. Retrospective studies suggest a higher prevalence of extensive CAD in these patients compared to those with sustained ROSC after CCPR, indicating a potential benefit of early revascularisation [3, 17]. However, revascularisation and intensive anti-thrombotic therapy may increase the bleeding risk, a significant concern in patients receiving venoarterial extracorporeal membrane oxygenation (VA-ECMO) [15, 18]. There is currently no prospective evidence to guide CAG and revascularisation strategy in ECPR patients, and the diagnostic value of ECG in this context remains unclear. This study aimed to evaluate post-cannulation ECPR management, focusing on coronary workup and the diagnostic value of the ECG.

## Methods

### Study design and population

We conducted a single-centre, retrospective chart review at Amsterdam University Medical Centre (Amsterdam UMC), including all consecutive adult CA patients who received ECPR through December 2024 and met the study inclusion criteria. Eligible patients were adults (≥ 18 years) who received ECPR, defined by the Extracorporeal Life Support Organisation (ELSO) definition as VA-ECMO in CA patients in whom CCPR fails to achieve sustained ROSC (for ≥ 20 consecutive minutes) [19, 20]. Patients with intermittent ROSC were included if cannulation began within 20 min of ROSC. Those cannulated after sustained ROSC were excluded. Both in-hospital cardiac arrest (IHCA) and out-of-hospital cardiac arrest (OHCA) patients were eligible.

### ECPR program

The Amsterdam UMC ECPR program was initiated in 2018. Patients with refractory CA were eligible for ECPR if the arrest was witnessed, CPR was initiated within three minutes, and there was either an initial shockable rhythm or a non-shockable rhythm with a suspected reversible cause (i.e., pulmonary embolism, intoxication, or hypothermia). Exclusion criteria included CPR duration > 45 min, age above 70 years, low pre-existing cognitive performance (Cerebral Performance Category [CPC] < 3), pre-existing heart- or pulmonary failure (NYHA class III/IV, COPD Gold class III/IV), history of metastatic or haematologic malignancy, history of bifemoral surgery, a live expectancy < 1 year, a do-not resuscitate policy or body mass index (BMI) > 40 mg/kg^2^. From June 2023 onward, following the INCEPTION trial, patients were excluded if aged over 60 years, had persistent asystole, or if cannulation could not be achieved within 45 min after cardiac arrest onset [12]. The local protocol recommends immediate CAG for those presenting with VF/VT, initial CT imaging of the thorax, abdomen, and brain, followed by targeted intervention as appropriate for those presenting with PEA, and rewarming in the operating theatre for those presenting with hypothermia. Eligibility and exclusion criteria, as well as initial protocolised management, are also listed in the Electronic Supplementary Material 1.

### Data acquisition

Baseline characteristics, medical history, and CAD risk factors were recorded, along with detailed information on the CA event, including location, witnessed status, time to CPR, mechanical CPR use, time of the CA, initial rhythm, intermittent ROSC, and refractory ventricular fibrillation (VF) or pulseless ventricular tachycardia (VT) (≥ 5 consecutive rhythms of VF/VT). Laboratory data, ECPR cannulation details, and key time intervals (CA event, emergency department arrival, VA-ECMO cannulation, flow initiation, and CAG initiation) were also collected. ECG data were collected at three time points: Prehospital, in-hospital before cannulation, and in-hospital after VA-ECMO cannulation but before CAG or diagnosis. ST-segment elevation was defined according to the Fourth Universal Definition of Myocardial Infarction and considered positive in case present on any of the three ECGs [21]. For those undergoing CAG, data on CAD, culprit lesion (either identified by the treating interventional cardiologist or as an acute [sub]total coronary occlusion on CAG with concomitant signs or a diagnosis of AMI documented in the chart), multivessel disease, chronic total occlusions (CTO), revascularisation by percutaneous coronary intervention [PCI] or coronary artery bypass grafting [CABG], the number of implanted stents and the TIMI Flow (range 0–3) before and after PCI. Additional imaging findings were also collected, as was the use of mechanical circulatory support (MCS) devices.

#### Statistical analysis

Continuous variables were described as mean ± standard deviation (SD) or median (interquartile range [IQRs]) and compared using a two-sample *t*- or Mann-Whitney U‑test. Categorical variables were presented as counts (percentages) and compared using the chi-square or Fisher’s exact test as appropriate. Missing data were excluded. Univariate logistic regression identified predictors of undergoing CAG and having a culprit lesion. Firth’s penalized likelihood logistic regression was used for complete separation. Kaplan-Meier survival curves were used to compared in-hospital mortality by CAG status, with the x‑axis truncated at 30 days for visualisation purposes only. Subgroup analyses included initial shockable rhythm and those with or without ST-segment elevation. Cox proportional hazards regression was used to estimate hazard ratios (HR) and 95% confidence intervals (CI). All statistical analyses were performed using R version 4.4.3 (R-packages: *tableone, logistf, survival).*

#### Ethical approval

This study was conducted in accordance with the Declaration of Helsinki (Finland, 2024) and reviewed by the Medical Ethics Review Committee of the Amsterdam UMC, which deemed it outside the scope of the Medical Research Involving Human Subject Act. Data usage consent was obtained via an opt-out procedure.

## Results

### Patients

**Fig. 1:**
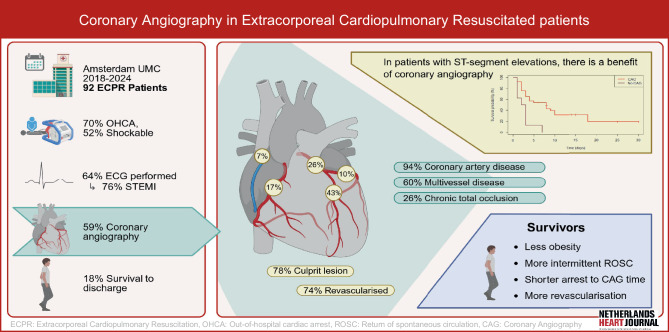
*Infographic:* Coronary Angiography in Extracorporeal Cardiopulmonary Resuscitation Patients

This study included 92 ECPR patients, with a mean age of 53 (SD 14) years, and of whom 60 (65%) were male. (Tab. 1, See Electronic Supplementary Material [ESM] Fig S1) Cardiovascular risk factors, including obesity, hypercholesterolemia, and hypertension, were present in 33%, 33%, and 43%, respectively. In 59 (64%) patients, an ECG was obtained, showing ST-segment elevation in 45 (76%). VA-ECMO flow following cannulation was established in 88 (96%) patients, and 14 (15%) were cannulated percutaneously. Periprocedural bleeding occurred in 10 (11%) patients (Tab. 1; Fig. 1).

**Table 1:** Baseline and admission characteristics

Baseline characteristics	Overall (*n* = 92)	Non-Survivors (*n* = 75)	Survivors (*n* = 17)	*p*
Age, years	53.3 (13.7)	53.6 (14.1)	52.1 (12.1)	0.686
Male sex	60/92 (65.2%)	50/75 (66.7%)	10/17 (58.8%)	0.741
Smoker	23/54 (42.6%)	16/39 (41%)	7/15 (46.7%)	0.946
Diabetes	14/84 (16.7%)	12/67 (17.9%)	2/17 (11.8%)	0.725
Obesity	27/83 (32.5%)	26/66 (39.4%)	1/17 (5.9%)	0.008
Hypercholesterolemia	27/81 (33.3%)	22/65 (33.8%)	5/16 (31.2%)	1.000
Hypertension	35/81 (43.2%)	30/65 (46.2%)	5/16 (31.2%)	0.426
Positive family history	13/45 (28.9%)	11/35 (31.4%)	2/10 (20.0%)	0.698
Congestive heart failure	13/84 (15.5%)	12/67 (17.9%)	1/17 (5.9%)	0.451
History of arrhythmia	15/84 (17.9%)	14/67 (20.9%)	1/17 (5.9%)	0.285
Antithrombotic agent(s)	25/83 (30.1%)	20/66 (30.3%)	5/17 (29.4%)	1.000
*Laboratory values on admission*				
Lactate, mmol/L	12.6 (4.4)	12.9 (4.2)	10.8 (5.2)	0.144
pH	6.88 (0.23)	6.87 (0.22)	6.94 (0.28)	0.321
Haemoglobin (mmol/L)	8.1 [6.7–9.0]	8.1 [6.9–8.9]	8.2 [5.58–9.28]	0.822
Leukocytes (10^9/L)	11.40 [8.45, 15.85]	11.40 [8.50, 15.65]	11.45 [7.17, 19.10]	0.759
Creatinine (μmol/L)	108.1 (29.6)	109.8 (29.9)	98.8 (27.2)	0.262
Glucose (mmol/L)	19.1 (8.6)	19.4 (8.3)	17.3 (10.1)	0.417
*Extracorporeal cardiopulmonary resuscitation*				
Percutaneous cannulation	14/92 (15.2%)	10/75 (13.3%)	4/17 (23.5%)	0.283
Periprocedural bleeding	10/92 (10.9%)	7/75 (9.3%)	3/17 (17.6%)	0.386
Wrong cannula position	7/92 (7.6%)	5/75 (6.7%)	2/17 (11.8%)	0.609
Flow established	88/92 (95.7%)	71/75 (94.7%)	17/17 (100%)	1.000
*Diagnostic workup*				
ECG*	59/92 (64.1%)	45/75 (60%)	14/17 (82.4%)	0.099
– ST-segment elevation	45/59 (76.3%)	34/45 (75.6%)	11/14 (78.6%)	1.000
Quick look TTE	83/91 (91.2%)	69/75 (92%)	14/16 (87.5%)	0.626
– LVEF < 25%	40/55 (72.7%)	33/47 (70.2%)	7/8 (87.5%)	0.423
– Dilated right ventricle	16/70 (22.9%)	14/58 (24.1%)	2/12 (16.7%)	0.720
– Pericardial effusion	5/74 (6.8%)	4/62 (6.5%)	1/12 (8.3%)	1.000
Computer Tomography	37/92 (40.2%)	32/75 (42.7%)	5/17 (29.4%)	0.464
Coronary Angiography	54/92 (58.7%)	43/75 (57.3%)	11/17 (64.7%)	0.776
*Additional mechanical support during admission*				
IABP	6/92 (6.5%)	4/75 (5.3%)	2/17 (11.8%)	0.306
Microaxial flow pump	15/92 (16.3%)	13/75 (17.3%)	2/17 (11.8%)	0.729

*ECG* electrocardiogram, *TTE* transthoracic echocardigraphy, *LVEF* left ventricular ejection fraction, *IABP* intra-aortic balloon pump

**Fig. 2:**
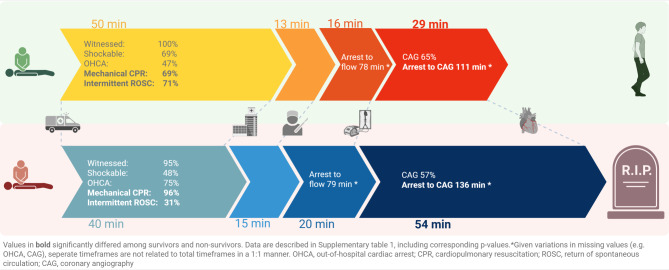
Arrest specifics and timing intervals of survivors and non-survivors

Most CAs concerned OHCAs (*n* = 64, 70%) and almost all were witnessed (*n* = 88, 96%). An initial shockable rhythm was present in 47 (52%) patients, of whom 40 (85%) underwent CAG. Among the 44 patients with initial non-shockable rhythm, 14 (32%) underwent CAG, resulting in a total of 54 patients (59%) receiving CAG (Figs. 1 and 2, ESM Tab. S1 and ESM Tab. S2).

### Survivors versus non-survivors

Seventeen patients (18%) survived to discharge. Survivors were less often obese (6% vs. 39%, *p* = 0.019), more likely to have intermittent ROSC (71% vs. 31%, *p* = 0.005), and less likely to have received mechanical compressions (69% vs. 96%, *p* = 0.005) (Tab. 1; Fig. 2, and ESM Tab S1). Median time from CA onset to VA-ECMO flow was 79 (62–92) minutes, with no difference between survivors and non-survivors (Fig. 2, ESM Tab S1). Survival to discharge was 11% in patients with initial non-shockable rhythm and 23% in those with an initial shockable rhythm (*p* = 0.218). Annual survival to discharge rates are shown in ESMTable S3.

### CAG and revascularisation

CAG was performed in 54 (59%) patients. Among these, significant CAD was present in 49 (94%), multivessel disease in 31 (60%), and at least one CTO in 14 (26%). Survivors had a shorter time to CAG (111 vs. 136 min, *p* = 0.026) (Tab. 2). A culprit lesion was identified in 42 (78%) patients undergoing CAG, most commonly in the anterior descending coronary artery (LAD) (*n* = 18, 43%), followed by the left main (LM) (*n* = 11, 26%), the right coronary artery (*n* = 7, 17%), the circumflex (*n* = 4, 10%) and a CABG-graft (*n* = 3, 7%).

**Table 2:** Coronary angiography

Coronary angiography	Overall (*n* = 54)	Non-Survivors (*n* = 43)	Survivors (*n* = 11)	*p*
*Before coronary angiography*				
Time arrest to CAG, min	128 [99, 155]	136 [107, 156]	111 [−16.50, 115]	0.026
Any ECG	42/54 (77.8%)	31/43 (72.1%)	11/11 (100%)	0.097
– ST-segment elevations	37/42 (88.1%)	26/31 (83.9%)	11/11 (100%)	0.303
Initial rhythm shockable	40/54 (74.1%)	30/43 (69.8%)	10/11 (90.9%)	0.253
*Coronary angiography findings*				
CAD severity				0.858
– No significant CAD	3/52 (5.8%)	3/42 (7.1%)	0/10 (0%)	
– Single VD	18/52 (34.6%)	15/42 (35.7%)	3/10 (30%)	
– Multiple VD	31/52 (59.6%)	24/42 (57.1%)	7/10 (70%)	
≥ 1 CTO	14/54 (25.9%)	11/43 (25.6%)	3/11 (27.3%)	1.000
Culprit coronary artery	42/54 (77.8%)	32/43 (74.4%)	10/11 (90.9%)	0.421
– LM	11/42 (26.2%)	8/32 (25%)	3/10 (30%)	1.000
– LAD	18/42 (42.9%)	13/32 (40.6%)	5/10 (50%)	0.875
– LCX	4/42 (9.5%)	4/32 (12.5%)	0/10 (0%)	0.557
– RCA	7/42 (16.7%)	6/32 (18.8%)	1/10 (10%)	1.000
– CABG-graft	3/42 (7.1%)	2/32 (6.2%)	1/10 (10%)	1.000
Revascularized	40/54 (74.1%)	29/43 (67.4%)	11/11 (100%)	0.048
TIMI-pre PCI ≤ 1	27/37 (73.0%)	19/28 (67.9%)	8/9 (88.9%)	0.393
TIMI-post PCI ≥ 2	36/37 (97.3%)	27/28 (96.4%)	9/9 (100%)	1.000

*CAG* coronary angiography, *ECG* electrocardiogram, *CAD* coronary artery disease, *VD* vessel disease, *CTO* chronic total occlusion, *LM* left main, *LAD* left anterior descending artery, *LCX* left circumflex artery, *RCA* right coronary artery, *CABG* coronary artery bypass graft, *TIMI* Thrombolysis In Myocardial Infarction, *PCI* percutaneous coronary intervention

Revascularisation was performed in 40 (74%) patients who underwent CAG, and TIMI‑2 or -3 flow post-revascularisation was achieved in 97% of cases. All patients (*n* = 11) who underwent CAG and survived were revascularised, whereas revascularisation was performed in only 29 (67%) of the non-survivors (*p* = 0.048) (Tab. 2). In three cases, revascularisation was withheld despite a culprit lesion. All three died from refractory cardiogenic shock. Two had an occluded CABG-graft in the context of significant multivessel disease, and the third case had a papillary muscle rupture following a prior circumflex infarction.

### Survival with and without CAG

**Fig. 3:**
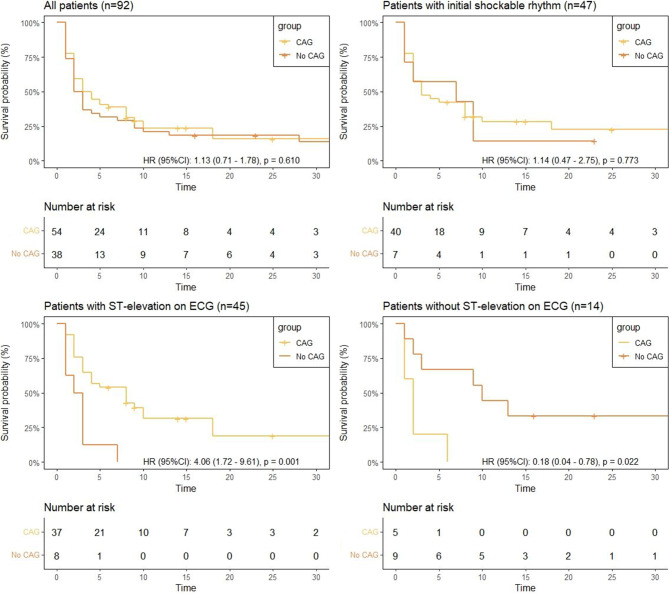
Kaplan Meier curves—CAG versus no CAG in various subcohorts

CAG was not associated with higher survival rates in the overall cohort (*n* = 92), or among patients with an initial shockable rhythm (*n* = 47). Among 45 patients with ST-segment elevations, all 8 who did not undergo CAG died, indicating a significantly higher risk of in-hospital mortality (HR 4.06, 95%CI 1.72–9.61) (Fig. 3) CAG was mainly withheld due to alternative diagnoses (e.g., pulmonary embolism) (ESM Tab S4). Among those without ST-segment elevations (*n* = 14), all 5 patients who underwent CAG died, and not undergoing CAG was associated with a lower in-hospital mortality risk (HR 0.18, 95%CI 0.04–0.78) (Fig. 3).

### Predictors of undergoing CAG and having a culprit

Univariate analyses showed that male sex (OR 4.83), initial shockable rhythm (OR 12.24), refractory VF/VF (OR 59.40), number of defibrillations (OR 1.59 per defibrillation), ECG obtained (OR 4.32), and ST-segment elevations on ECG (OR 8.32) were associated with undergoing CAG. Right-ventricle dilation and/or pericardial effusion on echo were negatively associated. Among CAG patients, a culprit lesion was less often found in patients with hypercholesterolemia (OR 0.21), a history of congestive heart failure (OR 0.19), a history of arrhythmias (OR 0.13), and those using antithrombotic medication (OR 0.18) (Tab. 3).

**Table 3:** Factors associated with undergoing coronary angiography and having a culprit lesion

	CAG performed		Culprit on CAG	
	OR	95%CI	OR	(95%CI)
*Patient characteristics*				
– Sex—Male	4.83	1.96–12.51	0.29	0.01–1.79
– Hypercholesterolemie	1.90	0.73–5.30	0.21	0.05–0.81
*Cardiac history*				
– History of congestive HF	0.98	0.30–3.54	0.19	0.04–0.96
– History of arrythmia	0.91	0.29–2.98	0.13	0.02–0.61
– Anti-thrombotic medication before admission	1.30	0.49–3.64	0.18	0.04–0.71
*Arrest specifics*				
– Shockable first rhythm	12.24	4.62–36.38	0.94	0.18–3.85
– Refractory VF/VT (> 5 shockblocks)	59.40	11.43–1096	0.78	0.18–2.93
– Number of defibrillations	1.59	1.31–2.07	0.91	0.80–1.04
*Diagnostisc workup*				
– Any ECG	4.32	1.78–10.99	2.13	0.47–8.74
– ST-segment elevation on ECG	8.32	2.29–34.14	3.44	0.39–25.64
– TTE: RV-dilatation (Firth)	0.01	0.00–0.08	–	–
– TTE: Pericardial effusion (Firth)	0.06	0.00–0.52	–	–
– CT-scan performed	0.18	0.07–0.45	0.94	0.23–4.83

*HF* heart failure, *VF* ventricular fibrillation, *VT* ventricular tachycardia, *TTE* transthoracic echocardiography, *RV* right ventricle, *CT* computer tomography

*tested but not associated: Age, hypertension, obesity, diabetes, active smoker, positive family history, OHCA/IHCA, LVEF below 25%, creatine, leucocytes

## Discussion

This study evaluated CAG use and the role of ECG findings in ECPR patients. Among those undergoing CAG, nearly all had significant CAD, three-quarters had a culprit lesion, almost two-thirds had multivessel disease, and a quarter had at least one CTO. Prehospital or early ECG was available in only two-thirds of patients, and the benefit of CAG differed between those with and without ST-segment elevations.

The reasons for the absence of an ECG remain uncertain and may be due to the lack of organised cardiac rhythm, immediate transfer for further procedures (e.g., catheterisation laboratory, CT scanner, or operating theatre), or unarchived ECGs. Ongoing resuscitation prior to cannulation may also deprioritise ECG acquisition following the restoration of an organised cardiac rhythm. After VA-ECMO cannulation, improved coronary perfusion may increase the likelihood of regaining an organised cardiac rhythm [22, 23]. Defibrillation shortly after VA-ECMO is therefore reasonable and, if an organised, interpretable rhythm is restored, ECG acquisition is crucial, as this yields additional diagnostic information. The presence of ST-segment elevations may strongly indicate immediate CAG, perhaps even in the presence of an alternative diagnosis [24]. In the absence of ST-segment elevations, the role of immediate CAG is less clear. The observed higher mortality in those without ST-elevations undergoing CAG may reflect a selected, sicker subpopulation rather than harmful effects of the angiography itself. Importantly, CAG will always provide full information on the coronary status, which may be important for further clinical decision-making, including in those without ST-elevation. Although further research is needed to clarify the role of revascularisation in weaning from VA-ECMO, previous evidence has demonstrated a clear benefit of revascularisation in cardiogenic shock patients [25]. Future research is needed in this field to determine the optimal strategy.

CAG was performed in 59% of the cases and showed high rates of complex CAD, which could explain the refractory nature of the CA in these patients [17, 26–28]. The decision to perform CAG was associated with sex, initial rhythm, number of defibrillations, ECG findings, and quick-look echo results. Signs suggestive of myocardial ischemia (e.g., persistent VF/VT and ST-segment elevation) increased the likelihood of CAG, while signs of alternative diagnoses (e.g., right-ventricle dilation) reduced it. Although reasonable, one could argue that knowing the complete coronary status in patients with refractory CA is important for further clinical management. In some cases, pulmonary embolism precluded CAG, whereas autopsy later showed the presence of a coronary thrombus. Notably, all patients with ST-segment elevation who did not undergo CAG died, emphasizing the importance of not ruling out a cardiac cause too quickly. Interestingly, cardiac risk factors or comorbidities like hypercholesterolemia, congestive heart failure, arrhythmia, and the use of anti-thrombotic medication were associated with lower odds of a culprit lesion, possibly due to old arrhythmic substrates or a lower threshold for CAG in these patients. Although counterintuitive, this finding is underscored by recent literature [29].

In-hospital survival was 18%, which is consistent with some literature, yet lower than the ± 30% reported in contemporary ELSO Registry data. [12, 13, 29–31]. This may be explained by several factors, including patient selection, logistical factors and the timing of VA-ECMO. Approximately half of the patients had an initial non-shockable rhythm. Despite a suspected reversible cause being required for ECPR initiation, survival to discharge in the non-shockable cohort was only 11%. In addition, the ECPR program at Amsterdam UMC only commenced in 2018; both the initial learning curve and the COVID-19 pandemic may have influenced outcomes. Although the overall time from CA- to VA-ECMO flow was long in our cohort, this did not differ between survivors and non-survivors. Survivors did have shorter times from CA to CAG and from VA-ECMO flow to CAG. Notably, survivors more often experienced intermittent ROSC, supporting recent literature that patients with periods of restored circulation have better outcomes than those in continuous CA [30]. Although intuitive, this finding may be valuable in guiding decisions on whether to initiate ECPR. Intermittent ROSC may also explain the relatively long CA- to VA-ECMO flow time in survivors. Additionally, obesity, which is often considered a relative contraindication to ECPR, was more prevalent among non-survivors [15].

The aim of this study was to evaluate post-cannulation ECPR management, focusing on coronary workup and the diagnostic value of the ECG. Our findings underscore the importance of routine ECG assessment as part of the post-cannulation work-up in all ECPR patients. Given the high prevalence of CAD, a comprehensive coronary workup with CAG, especially in those with ST-segment elevations, is also recommended. Further studies are necessary to determine the role of immediate CAG in patients without ST-segment elevations.

### Limitations

This study has several limitations. The small sample size limits statistical power and precludes multivariable analysis. Associations are unadjusted, and confidence intervals are wide. The results should therefore be interpreted in light of potential confounding. The retrospective, single-centre design introduces selection and information bias, particularly because ECGs and CAGs were not systematically obtained in all patients. This may limit generalisability and highlights a knowledge gap that should be systematically addressed in future research.

## Conclusion

In ECPR patients, CAG revealed a high prevalence of significant CAD and culprit lesions. Pre-hospital or early ECG was obtained in only 64% of patients. The benefit of CAG appeared to differ by ECG findings, being most evident in those with ST-segment elevations, underscoring the importance of ECG acquisition. Given the high prevalence of culprit lesions, a comprehensive coronary workup with CAG is recommended, especially in those with ST-segment elevations. Further studies are necessary to determine the role of immediate CAG in patients undergoing ECPR without ST-segment elevations.

## Supplementary Information

ESM1: Supplementary material 1

## Data Availability

Data can be made available upon reasonable request.
