# Supplementary material for: Coronary angiography in cardiac arrest patients undergoing extracorporeal cardiopulmonary resuscitation
Source: Neth Heart J. 2026 May 18;34(6):216–24. doi: 10.1007/s12471-026-02049-3 (PMC13216418; doi:10.1007/s12471-026-02049-3)
Supplement: Supplementary file 1 — ESM1: Supplementary material 1 [file 12471_2026_2049_MOESM1_ESM.docx]

**Supplemental material**

Supplement 1. ECPR Program Amsterdam UMC: In- and exclusion criteria and initial workflow

Figure S1. Flowchart

Table S1. Data accompanying Figure 2

Table S2. Imaging modalities plus findings, and survival to discharge according to initial rhythm

Table S3. Annual survival to discharge

Table S4. Reasons no coronary angiography was performed in patients with ST-segment elevation

**Supplement 1. ECPR Program Amsterdam UMC: In- and exclusion criteria and initial workflow**

Patients with refractory cardiac arrest were eligible for ECPR if:

- The cardiac arrest was witnessed, AND
- CPR was initiated within 3 minutes, AND
- The initial rhythm was shockable, OR non-shockable with a suspected aetiology of pulmonary embolism, intoxication, or hypothermia

Exclusion criteria:

- CPR duration > 45 minutes
- Age > 70 years (≥ 60 years after June 2023)
- Pre-existing Cerebral Performance Category (CPC) ≥ 3
- COPD GOLD class III/IV
- Heart failure NYHA class III/IV
- Metastatic or haematologic malignancy
- History of bifemoral surgery
- Life expectancy <1 year
- Do-not-resuscitate policy
- BMI > 40 mg/kg^2^
- Inability to initiate cannulation within 45 minutes of arrest onset (after June 2023)
- Persistent asystole (after June 2023)

**Protocolised Management**

- Patients with VF/VT undergo direct CAG
- Patients with PEA undergo initial CT-imaging of the thorax, abdomen, and brain, followed by targeted intervention as indicated
- Hypothermic patients are rewarmed in the operating theatre

**Figure S1. Flowchart**


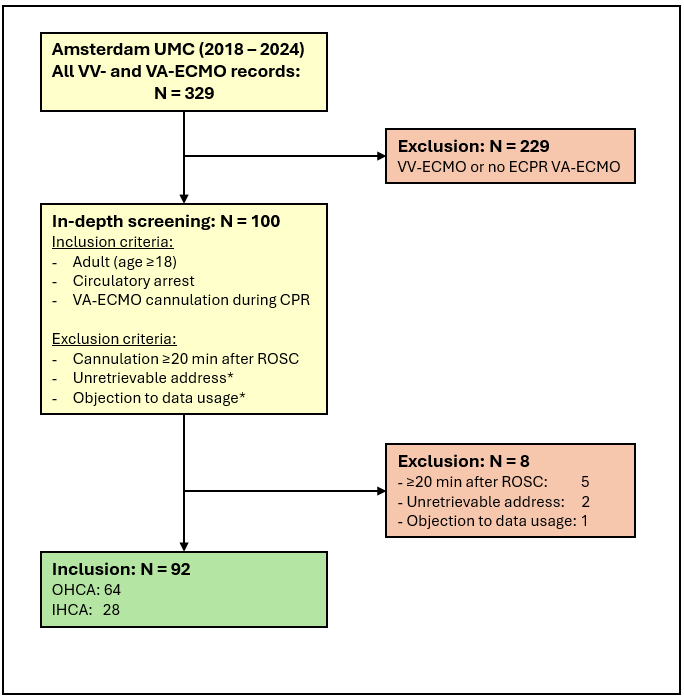


UMC, university medical centre; VV, venovenous; VA-ECMO; venoarterial extracorporeal membrane oxygenation, (E)CPR, (extracorporeal) cardiopulmonary resuscitation; ROSC, return of spontaneous circulation; OHCA, out-of-hospital cardiac arrest; IHCA, in-hospital cardiac arrest.

**Table S1. Data input Figure 1**

| **Baseline characteristics** | **Overall (n=92)** | **Non-Survivors (n=75)** | **Survivors (n=17)** | **p** |
| --- | --- | --- | --- | --- |
| Specifics cardiac arrest |  |  |  |  |
| Witnessed | 88/92 (95.7%) | 71/75 (94.7%) | 17/17 (100%) | 1.000 |
| Initial rhythm |  |  |  | 0.454 |
| Asystole | 8/91 (8.8%) | 7/75 (9.3%) | 1/16 (6.2%) |  |
| PEA | 36/91 (39.6%) | 32/75 (42.7%) | 4/16 (25%) |  |
| pulseless VT | 4/91 (4.4%) | 3/75 (4%) | 1/16 (6.2%) |  |
| VF | 43/91 (47.3%) | 33/75 (44%) | 10/16 (62.5%) |  |
| Shockable | 47/91 (51.6%) | 36/75 (48%) | 11/16 (68.8%) | 0.218 |
| Refractory VF/VT | 34/90 (37.8%) | 26/74 (35.1%) | 8/16 (50%) | 0.408 |
| OHCA | 64/92 (69.6%) | 56/75 (74.7%) | 8/17 (47.1%) | 0.052 |
| IHCA location |  |  |  | 0.779 |
| General ward | 5/28 (17.9%) | 4/19 (21.1%) | 1/9 (11.1%) |  |
| CCU | 3/28 (10.7%) | 2/19 (10.5%) | 1/9 (11.1%) |  |
| ICU | 4/28 (14.3%) | 3/19 (15.8%) | 1/9 (11.1%) |  |
| OR | 5/28 (17.9%) | 3/19 (15.8%) | 2/9 (22.2%) |  |
| Cath-lab | 8/28 (28.6%) | 4/19 (21.1%) | 4/9 (44.4%) |  |
| ED | 3/28 (10.7%) | 3/19 (15.8%) | 0/9 (0%) |  |
| Time CPR |  |  |  | 0.664 |
| Immediate | 58/90 (64.4%) | 46/74 (62.2%) | 12/16 (75%) |  |
| 1-5 minutes | 25/90 (27.8%) | 22/74 (29.7%) | 3/16 (18.8%) |  |
| 6-10 minutes | 4/90 (4.4%) | 3/74 (4.1%) | 1/16 (6.2%) |  |
| >11 minutes | 3/90 (3.3%) | 3/74 (4.1%) | 0/16 (0%) |  |
| Mechanical compressions | 76/84 (90.5%) | 65/68 (95.6%) | 11/16 (68.8%) | 0.005 |
| Intermittent ROSC | 35/92 (38%) | 23/75 (30.7%) | 12/17 (70.6%) | 0.005 |
| Total time ROSC, minutes (median [IQR]) | 10 [5-21] | 10 [8-17] | 10.5 [4-28.25] | 0.692 |
| CAG performed | 54/92 (58.7%) | 43/75 (57.3%) | 11/17 (64.7%) | 0.776 |
| CAG before cannulation | 9/54 (16.7%) | 5/43 (11.6%) | 4/11 (36.4%) | 0.072 |
| Time intervals |  |  |  |  |
| Arrest to ED (median [IQR]) | 40 [32.25-50] | 39.5 [32-48.5] | 50 [39.25-60.75] | 0.156 |
| ED to cannulation (median [IQR]) | 15 [9.25-21.75] | 15 [10-24] | 13 [4-17] | 0.285 |
| Cannulation to flow (median [IQR]) | 19 [14.25-25.75] | 19.5 [15-26.75] | 15.5 [12.25-18.5] | 0.139 |
| Flow to CAG (median [IQR]) | 49 [25-65] | 54 [28.25-69.5] | 29 [-99-50] | 0.048 |
| Arrest to flow (median [IQR]) | 78.5 [62.25-91.5] | 79 [63.5-92.5] | 78 [60.5-83] | 0.333 |
| Arrest to CAG (median [IQR]) | 128 [99-155] | 136 [107-156] | 111 [-16.5-115] | 0.026 |

PEA, pulseless electrical activity; VT, ventricular tachycardia; VF, ventricular fibrillation; OHCA, out-of-hospital cardiac arrest; MMT, Mobile Medical Team. IHCA, in-hospital cardiac arrest; CCU, coronary care unit; ICU, Intensive Care Unit; OR, operation room; ED, emergency department; CPR, cardiopulmonary resuscitation; ROSC, return of spontaneous circulation; CAG, coronary angiography.

**Table S2. Imaging modalities plus findings, and survival to discharge according to initial rhythm**

|  | **All patients (n=92)** | **Non-shockable (n=44)** | **Shockable (n=47)** | **p** |
| --- | --- | --- | --- | --- |
| Imaging |  |  |  |  |
| ECG | 59/92 (64.1%) | 27/44 (61.4%) | 32/47 (68.1%) | 0.652 |
| ST-segment elevation | 45/59 (76.3%) | 18/27 (66.7%) | 27/32 (84.4%) | 0.199 |
| Quick look TTE | 83/91 (91.2%) | 41/44 (93.2%) | 42/46 (91.3%) | 1.000 |
| LVEF <25% | 40/55 (72.7%) | 14/23 (60.9%) | 26/32 (81.2%) | 0.172 |
| Dilated right ventricle | 16/70 (22.9%) | 16/34 (47.1%) | 0/36 (0%) | <0.001 |
| Pericardial effusion | 5/74 (6.8%) | 4/38 (10.5%) | 1/36 (2.8%) | 0.358 |
| Computer tomography | 37/92 (40.2%) | 23/44 (52.3%) | 13/47 (27.7%) | 0.029 |
| Head CT | 31/92 (33.7%) | 21/44 (47.7%) | 10/47 (21.3%) | 0.015 |
| Findings |  |  |  | 0.945 |
| No relevant abnormalities | 19/31 (61.3%) | 12/21 (57.1%) | 7/10 (70%) |  |
| Intracranial bleeding | 1/31 (3.2%) | 1/21 (4.8%) | 0/10 (0%) |  |
| Intracranial ischemia | 2/31 (6.5%) | 2/21 (9.5%) | 0/10 (0%) |  |
| Cerebral swelling | 6/31 (19.4%) | 4/21 (19%) | 2/10 (20%) |  |
| Post anoxic brain injury | 3/31 (9.7%) | 2/21 (9.5%) | 1/10 (10%) |  |
| CT-thorax/abdomen | 34/92 (37%) | 23/44 (52.3%) | 11/47 (23.4%) | 0.009 |
| Findings |  |  |  | <0.001 |
| No relevant abnormalities | 18/34 (52.9%) | 9/23 (39.1%) | 9/11 (81.8%) |  |
| Pulmonary embolism | 14/34 (41.2%) | 14/23 (60.9%) | 0/11 (0%) |  |
| (Tension) pneumothorax | 2/34 (5.9%) | 0/23 (0%) | 2/11 (18.2%) |  |
| Coronary angiography | 54/92 (58.7%) | 14/44 (31.8%) | 40/47 (85.1%) | <0.001 |
| Culprit identified | 42/54 (77.8%) | 11/14 (78.6%) | 31/40 (77.5%) | 1.000 |
| revascularised | 40/54 (74.1%) | 10/14 (71.4%) | 30/40 (75%) | 1.000 |
| Clinical outcome |  |  |  |  |
| Survival to discharge | 17/92 (18.5%) | 5/44 (11.4%) | 11/47 (23.4%) | 0.218 |

ECG, electrocardiogram; TTE, transthoracic echocardiography; LVEF, left ventricular ejection fraction; CT, computer tomography.

**Table S3. Annual survival to discharge**

| **Year of ECPR** | **Survival to discharge (%)** |
| --- | --- |
| 2018 | 1/8 (12.5%) |
| 2019 | 0/10 (0.0%) |
| 2020 | 3/14 (21.4%) |
| 2021 | 4/15 (26.7%) |
| 2022 | 4/25 (16.0%) |
| 2023 | 1/9 (11.1%) |
| 2024 | 4/11 (36.4%) |

**Table S4. Reasons no coronary angiography was performed in patients with ST-segment elevation**

| **#** | **ST-segment prehospital** | **ECG before cannulation** | **ECG after cannulation** | **Computer Tomography** | **Reason no coronary angiography was performed** |
| --- | --- | --- | --- | --- | --- |
| 1 | x | x | ST-segment elevation | Pulmonary embolism | Thrombolysis for pulmonary embolism. PCI deemed unnecessary by treating physician. Acute coronary occlusion identified during autopsy |
| 2 | ST-segment elevation | Nonspecific ST-deviations | Nonspecific ST-deviations | No relevant abnormalities | Primary cardiac cause deemed unlikely based on in-hospital ECGs |
| 3 | ST-segment elevation | Nonspecific ST-deviations | x | Pulmonary embolism and post-anoxic brain injury | Presence of an alternative diagnosis (pulmonary embolism) |
| 4 | x | ST-segment elevation | x | no CT | Intrathoracic and myocardial haemorrhage post cardiotomy, went for re-thoracotomy. Treatment discontinued in the OR |
| 5 | Nonspecific ST-deviations | x | ST-segment elevation | Pulmonary embolism | Presence of an alternative diagnosis (pulmonary embolism) |
| 6 | x | ST-segment elevation | ST-segment elevation | No relevant abnormalities | Coagulopathy with ongoing bleeding after VA-ECMO cannulation, deceased shortly after ECMO. Posterior wall infarction and coronary microthrombi identified during autopsy. |
| 7 | ST-segment elevation | x | Iso-electric ST-segments | Pulmonary embolism | Presence of an alternative diagnosis (pulmonary embolism) and severe coagulopathy |
| 8 | x | ST-segment elevation | x | Pulmonary embolism | Presence of an alternative diagnosis (pulmonary embolism). ST-segment elevations considered post-resuscitation |
